# Novel Candidate Genes for Non-Syndromic Tooth Agenesis Identified Using Targeted Next-Generation Sequencing

**DOI:** 10.3390/jcm11206089

**Published:** 2022-10-15

**Authors:** Barbara Biedziak, Ewa Firlej, Justyna Dąbrowska, Agnieszka Bogdanowicz, Małgorzata Zadurska, Adrianna Mostowska

**Affiliations:** 1 Department of Orthodontics and Craniofacial Anomalies, Poznan University of Medical Sciences, 60-812 Poznan, Poland; 2 Department of Biochemistry and Molecular Biology, Poznan University of Medical Sciences, 61-781 Poznan, Poland; 3 Department of Orthodontics, Institute of Dentistry, Medical University of Warsaw, 02-091 Warszawa, Poland

**Keywords:** tooth agenesis, hypodontia, oligodontia, NGS panel, pathogenic variant

## Abstract

Non-syndromic tooth agenesis (ns-TA) is one of the most common dental anomalies characterized by the congenital absence of at least one permanent tooth (excluding third molars). Regarding the essential role of genetic factors in ns-TA aetiology, the present study aimed to identify novel pathogenic variants underlying hypodontia and oligodontia. In a group of 65 ns-TA patients and 127 healthy individuals from the genetically homogenous Polish population, the coding sequences of 423 candidate genes were screened using targeted next-generation sequencing. Pathogenic and likely pathogenic variants were identified in 37 (56.92%) patients, including eight nucleotide alternations of genes not previously implicated in ns-TA (*CHD7*, *CREBBP*, *EVC*, *LEF1*, *ROR2*, *TBX22* and *TP63*). However, since only single variants were detected, future research is required to confirm and fully understand their role in the aetiology of ns-TA. Additionally, our results support the importance of already known ns-TA candidate genes (*AXIN2*, *EDA*, *EDAR*, *IRF6*, *LAMA3*, *LRP6*, *MSX1*, *PAX9* and *WNT10A*) and provide additional evidence that ns-TA might be an oligogenic condition involving the cumulative effect of rare variants in two or more distinct genes.

## 1. Introduction

Odontogenesis is a complex and strictly regulated process that requires a series of sequential and reciprocal epithelial-mesenchymal cell interactions mediated by conserved signal transduction pathways, including Wnt/β-catenin, Bmp, Fgf, Shh and Eda [1,2]. Since these networks of signalling molecules and transcription factors play essential and widespread roles during embryogenesis, dentition provides an easily accessible and potentially general model to study organ development and regeneration [2]. Moreover, various anomalies in teeth size, shape, number and structure may be associated with defects in other tissues and organs [2]. For example, tooth agenesis (TA), which is a component of many clinically recognizable syndromes and multisystem disorders, including hypohidrotic ectodermal dysplasia (OMIM # 305100), Kallmann syndrome (OMIM # 147950), odonto-onycho-dermal dysplasia (OMIM # 257980), Witkop syndrome (OMIM # 189500) and van der Woude syndrome (OMIM # 119300). The congenital lack of teeth is also observed as an isolated condition (non-syndromic TA, ns-TA) and is classified as hypodontia or oligodontia based on the number of missing teeth. In most studies, the prevalence of hypodontia (agenesis of one to five teeth, excluding third molars) ranges from 3% to 10%, while the incidence of oligodontia (agenesis of 6 or more teeth, excluding third molars) varies from 0.1% to 0.5% [3]. The lack of wisdom teeth is more common and occurs in up to 30% of the general population [4].

The aetiology of ns-TA is multifactorial, with genetic and environmental factors considered possible contributing agents [3]. It has been estimated that in about 80% of affected individuals, ns-TA is caused by pathogenic variants of genes, the protein products of which are involved in craniofacial and tooth development [3]. In the remaining 20% of cases, ns-TA is attributed to exogenous factors, including trauma, chemotherapy and radiotherapy treatment in early infancy, as well as maternal smoking, viral infections, and medication intake during pregnancy [5]. In addition, Wang et al. have demonstrated that sporadic ns-TA might also be related to disturbances in DNA methylation patterns [6].

To date, numerous genes and nucleotide variants have been associated with the risk of ns-TA [3]. One of the key susceptibility genes for this dental anomaly is *WNT10A* (OMIM * 606268), which encodes a secreted signalling protein of the Wnt/β-catenin pathway involved in multiple stages of tooth development [7,8]. It has been demonstrated that more than half of individuals with hypodontia and oligodontia are carriers of the *WNT10A* risk variants, comprising the most frequently observed p.Phe228Ile (rs121908120) and p.Cys107Ter (rs121908119) [9]. The well-known genes implicated in ns-TA aetiology include also *AXIN2* (OMIM * 604025), *EDA* (OMIM * 300451), *EDAR* (OMIM * 604095), *LRP6* (OMIM * 603507), *MSX1* (OMIM * 142983) and *PAX9* (OMIM * 167416) [3,10]. It is worth noting that pathogenic variants of some of these genes are associated with syndromic forms of dental agenesis, suggesting that an apparent ns-TA exists either as a distinct entity or as part of a spectrum of syndromic conditions.

Recently, progress in identifying novel ns-TA risk variants has been made through next-generation sequencing (NGS) studies. Moreover, these investigations have demonstrated that in some familial cases, ns-TA might be an oligogenic condition involving the cumulative effect of more than one causal variant [11,12]. In most cases with suggestive evidence for a multilocus inheritance, one of the rare genetic alternations required for the phenotype expression is a missense or nonsense *WNT10A* variant [11,12,13,14,15,16]. These observations adding a layer of complexity to the genetic characterization of ns-TA might explain the inter or intrafamilial phenotypic variability, and apparent incomplete penetrance frequently observed in this dental anomaly [17].

The present retrospective study aimed to identify rare pathogenic variants underlying ns-TA by screening the coding sequences of 423 candidate genes using an NGS-based multi-gene panel testing in a group of 65 patients and 127 controls from the genetically homogenous Polish population.

## 2. Materials and Methods

### 2.1. Study Population

Peripheral blood samples were collected from 65 individuals with ns-TA (49.23% males) who were participants of the Comprehensive Therapy Programme for Craniofacial Anomalies at Poznan University of Medical Sciences. The median age of patients was 12 years (range from 7–17 years). The inclusion criterion was congenital agenesis of at least one permanent tooth, excluding third molars and lack of other apparent structural anomalies. The diagnosis of TA was based on clinical and panoramic radiographic examinations. Microdontia was not considered a form of tooth agenesis. Additionally, peg-shaped maxillary incisors were not considered as exclusion criterion in the current study. Case eligibility and non-syndromic designation were ascertained by clinicians, including an orthodontist, paediatrician, maxillofacial surgeon and speech therapist, using detailed diagnostic information from medical records. The patient group comprised 32 (49.23%) individuals with hypodontia and 33 (50.77%) individuals with oligodontia (Table 1). The median number of missing teeth per person was six (ranging from 1 to 24). The most commonly missing teeth were maxillary lateral incisors (16.63%), followed by mandibular second premolars (15.35%) and maxillary second premolars (11.94%). Tooth agenesis was associated with minor ectodermal features like fine hair, dry skin or brittle nails in eight patients (12.31%; seven with oligodontia and one with hypodontia). They were included in the study cohort since no known syndromes were diagnosed. A positive family history of ns-TA was reported by 36 (55.39%) study participants. Peripheral blood samples were also collected from 127 healthy individuals (45.67% males) with all permanent teeth present (not considering the third molars) and no family history of TA. All study participants were unrelated Caucasians of Polish origin. Genomic DNA was extracted from peripheral blood lymphocytes by salting-out extraction procedure. DNA concentration and quality were determined using a NanoDrop 2000 (Thermo Scientific, Wilmington, DE, USA). The study was conducted according to the Declaration of Helsinki [18] and was approved by the Institutional Review Board of Poznan University of Medical Sciences, Poland (approval number 1115/18). Written informed consent was obtained from all study participants or their legal guardians.

### 2.2. Next-Generation Sequencing (NGS)

The coding regions, including exon-intron boundaries of 423 genes, were screened in all patients and controls using targeted NGS to identify rare pathogenic variants associated with ns-TA. The multi-gene panel was developed previously to identify rare variants underlying craniofacial anomalies, including non-syndromic cleft lip with or without cleft palate (ns-CL/P). The detailed methodology for panel design, library preparation, NGS sequencing and data analysis has been described previously [19]. Briefly, the panel was created to target the coding sequence of genes associated with non-syndromic and syndromic forms of orofacial clefts (OFC) and TA and genes encoding proteins implicated in signalling pathways critical for craniofacial and orodental development. Detailed characteristics of all panel genes are presented in Table S1. The DNA probe set complementary to target regions with a total size of 1.59 Mb was designed using the NimbleDesign online tool (Roche, Madison, WI, USA). All sequencing libraries were constructed using the KAPA HyperPlus kit (Roche Sequencing Solutions, Pleasanton, CA, USA) and sequenced with 150-bp paired-end reads on a HiSeq 4000 platform (Illumina Inc., San Diego, CA, USA) according to the manufacturer’s protocol.

#### 2.2.1. Variant Detection and Annotation

The raw sequencing data were demultiplexed and converted to standard FASTQ format using bcl2fastq software (Illumina Inc., San Diego, CA, USA). After the quality control step, read sequences were aligned using the Burrows-Wheeler Aligner software (http://bio-bwa.sourceforge.net/, access on 1 March 2022) to the GRCh38/hg38 reference genome. Variant calling was conducted using the GATK tool (https://www.broadinstitute.org/gatk/, access on 1 March 2022) Identified variants were annotated with functional information, population frequency (http://gnomad.broadinstitute.org/, access on 1 March 2022) and association with clinically relevant phenotypes based on ClinVar (https://www.ncbi.nlm.nih.gov/clinvar/, access on 1 March 2022) and Human Gene Mutation Database (HGMD, http://www.hgmd.cf.ac.uk, access on 1 March 2022) online resources. Variant effects were predicted using 16 in silico algorithms, including MutationTaster, FATHMM, FATHMM-MKL, FATHMM-XF, LRT, DEOGEN2, EIGEN, EIGEN PC, SIFT, SIFT4G, PROVEAN, MVP, REVEL, PrimateAI, MetaSVM and MetaLR. In addition, the combined annotation dependent depletion (CADD) and adaptive boosting (ADA) scores were extracted from the genomic variant search engine VarSome (https://varsome.com/, access on 1 March 2022) for the missense variants and variants predicted to affect splicing.

#### 2.2.2. Variant Filtering and Prioritizing

To reduce the list of candidate variants for association with ns-TA, only exonic and splicing alternations (excluding synonymous) with a minor allele frequency (MAF) < 0.001 in the gnomAD European (non-Finnish) population were analyzed. The only exceptions were variants of the *WTN10A* gene, all of which were taken into evaluation. Stop-gain, stop-loss, frameshift, splicing with ADA score ≥ 0.9, and missense variants with CADD score > 20, which were designed as pathogenic/damaging by at least half of the in silico prediction algorithms, were included in likely gene disrupting variants. Variants identified within genes previously associated with the risk of non-syndromic or syndromic forms of TA, genes with a well-documented role in the odontogenesis and genes with a probability of being loss-of-function intolerant (pLi) score ≥ 0.95 were prioritized. The variants fulfilling the rigorous selection criteria were classified as pathogenic or likely pathogenic depending on whether they were annotated in the ClinVar or HGMD databases.

#### 2.2.3. Confirmation Analyses

The identified pathogenic and likely pathogenic variants were validated using Sanger sequencing, which was carried out using BigDyeTM Terminator Version 3.1 Ready Reaction Cycle Sequencing Kit on an ABI3730 Genetic Analyzer (Applied Biosystems, Waltham, CA, USA). The sequencing template PCR conditions and sequencing primers are presented in Table S2.

### 2.3. Common Variant Association Analysis

An analysis of common exonic and splicing alternations (MAF ≥ 0.1 in the case group) was conducted on genes (*n* = 16) with identified rare pathogenic and likely pathogenic variants. Their association with the risk of ns-TA was tested with the Cochran-Armitage trend test. The odds ratio (OR, allelic model) and corresponding 95% confidence intervals (95%CIs) were used to assess the strength of the association. *p*_trend_-values below 1.72 × 10^−3^ (0.05/29 tested variants) were interpreted as statistically significant.

## 3. Results

### NGS Analysis of 423-Panel Genes

After filtering, prioritization and validation steps, a final set of 27 pathogenic and likely pathogenic variants was identified. The detailed characteristics and in silico pathogenicity scores for all these genetic alternations identified in 37 ns-TA patients (56.92%) are presented in Table 2.

Eight likely pathogenic variants were detected in genes not previously correlated with the risk of ns-TA (Table 3, Table 4 and Table S3; Figures S1–S8), including one frameshift variant (EVC_p.Ala565ValfsTer23) and one nonsense variant (ROR2_p.Ser632Ter) predicted to cause nonsense-mediated mRNA decay (NMD). The other six genetic alterations were novel missense variants (CHD7_ p.Glu1856Gln, CBP_ p.Pro344Ser, CBP_ p.Glu1560Lys, LEF1_ p.Lys95Asn, TBX22_ p.Pro242Leu and TP63_ p.Pro532Ala) with a CADD score > 22 and classified as pathogenic or damaging/deleterious by most in silico prediction tools (Table 2). The only exception was the TP63_ p.Pro532Ala, which did not fulfil the project’s strict selection criteria (CADD = 18.76; verdict pathogenic/damaging by 7 out of 16 in silico tools). It was detected in a patient with hypodontia, abnormally shaped maxillary right lateral incisor and taurodontic maxillary first and second molars, who was diagnosed with complex odontoma at the age of 12 (Table 4). Three out of eight likely pathogenic variants identified in novel ns-TA candidate genes, including TP63_p.Pro532Ala, CBP_p.Glu1560Lys and EVC_p.Ala565ValfsTer23 were found in patients carrying one of the *WNT10A* missense alterations (Table 4).

**Figure 1:**
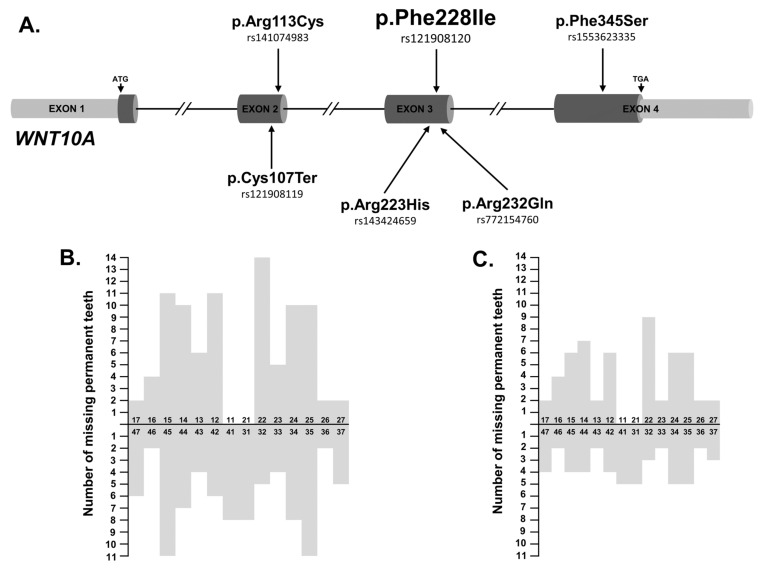
(**A**) Distribution of *WNT10A* nucleotide variants identified in ns-TA patients. (**B**) Number of missing permanent teeth in 18 patients with identified *WNT10* nucleotide variant/variants (*n* = 174, the mean number of missing permanent teeth per person, excluding third molars = 9.67, range 2–20). The most commonly missing teeth were second premolars, followed by lateral incisors and first premolars. (**C**) Number of missing permanent teeth in 9 patients exclusively harbouring the WNT10A_p.Phe228Ile variant (*n* = 104, the mean number of missing permanent teeth per person, excluding third molars = 11.6, range 3–20). The most commonly missing teeth were lateral incisors and first premolars followed by second premolars.

Six nucleotide variants detected in 18 ns-TA patients (27.69%) were known variants of the *WNT10A* gene (Table 2 and Table 4). All of them, except p.Arg113Cys, were changes with a CADD score > 22 and classified as pathogenic or damaging/deleterious by most in silico algorithms (Table 2). The p.Cys107Ter (rs121908119) and p.Phe228Ile (rs121908120) variants were reported in the ClinVar database as pathogenic alternations for selective TA (OMIM # 150400), Schopf-Schulz-Passarge syndrome (OMIM # 224750) and odontoonychodermal dysplasia (OMIM # 257980). In patients with the *WNT10A* variants, the mean number of missing permanent teeth per person, excluding third molars, was 9.67 (range 2–20). The most commonly missing teeth were second premolars, followed by lateral incisors and first premolars (Table 4 and Figure 1). A positive family history of TA was reported by 11 (61.11%) of these affected individuals.

The most frequently observed *WNT10A* variant in the patients’ group was the nonsynonymous substitution p.Phe228Ile. It was identified in either heterozygous or homozygous form in 15 ns-TA patients (MAF = 0.16), as well as in one healthy individual. Under an assumption of an allelic inheritance model, the calculated OR for the p.Phe228Ile variant was 47.86 (95%CI: 6.36–360.45, *p*_trend_ = 9.15 × 10^−7^; Table 5). In four patients harbouring the p.Phe228Ile substitution, another heterozygous *WNT10A* variant was also detected (Table 4). In addition, two ns-TA patients carrying this missense substitution had likely pathogenic variant identified in a gene not previously correlated with the risk of ns-TA, including *CREBBP* (OMIM * 600140) and *TP63* (OMIM * 603273) as described above (Table 4 and Figures S6 and S8).

The list of variants that met the selection criteria also comprised 13 variants of well-known ns-TA risk genes, such as *AXIN2*, *EDA*, *EDAR*, *IRF6* (OMIM * 607199), *LAMA3* (OMIM * 600805), *LRP6*, *MSX1* and *PAX9* (Table 2 and Table 3). The three top likely pathogenic missense changes with the highest CADD scores were EDA_ p.Arg289His (CADD = 31.0), IRF6_ p.Arg339Gly (CADD = 28.7) and LRP6_p.Arg473Pro (CADD = 25.0). All identified stop-gain variants (AXIN2_p.Arg675ProfsTer32, AXIN2_p.Val765LeufsTer24, LAMA3_p.Glu306Ter, MSX1_p.Leu123ThrfsTer52 and PAX9_p.Gln136Ter) were predicted to cause NMD. Two out of three *EDA* variants detected in male patients with hypodontia were classified by HGMD and ClinVar as pathogenic for ectodermal dysplasia (p.Arg69Pro; HGMD accession number CM960503) and pathogenic/likely pathogenic for hypohidrotic X-linked ectodermal dysplasia and X-linked selective TA (p.Arg289His; ClinVar interpretation). The heterozygous nonsense variant of the *LAMA3* gene identified in a patient with oligodontia (p.Glu306Ter, rs771405735) was reported in a ClinVar as a variant of uncertain significance for the autosomal recessive junctional epidermolysis bullosa gravis of Herlitz. The *PAX9* transition leading to premature stop codon formation (p.Gln136Ter) was described previously in a Finnish family with non-syndromic oligodontia [13]. The remaining nine variants were novel and not previously reported in databases or the available literature. The group of patients with identified variants in known ns-TA risk genes included four (28.57%) individuals with hypodontia and ten (71.43%) individuals with oligodontia. A family history of TA was reported by nine of them (64.29%).

Except for the WNT10A_p.Phe228Ile substitution, none of the identified pathogenic and likely pathogenic variants for ns-TA were detected in the control group. In addition, no variants that fulfilled the project’s selection criteria were detected in genes harbouring these coding variants in healthy individuals. The only exception was the *LAMA3* gene (pLi score of 0.00), in which a missense variant p.Asn2433Thr with a CADD score of 26 was identified in one control sample.

#### Association Analysis of Common Variants

Within genes harbouring pathogenic and likely pathogenic variants, 29 common exonic changes (11 missense and 18 silent) were identified (Table 5). Three of them were nominally associated with the risk of ns-TA in the tested population, including EVC_ p.Asn323Asn (*p*_trend_ = 4.90 × 10^−2^), IRF6_ p.Ser153Ser (*p*_trend_ = 3.29 × 10^−2^) and WNT10A_ p.Phe228Ile (*p*_trend_ = 9.15 × 10^−7^). The results for the latest variant, presented above, remained statistically significant even after adjusting for multiple comparisons.

## 4. Discussion

To investigate the role of rare nucleotide variants in the aetiology of ns-TA, a panel of functionally and clinically relevant candidate genes was analyzed using NGS in a group of 65 Polish patients with hypodontia and oligodontia. After applying a stringent variant filtering strategy, pathogenic and likely pathogenic nucleotide alternations were detected in 57% of affected individuals. Eight of these variants were identified in genes not previously associated with the ns-TA risk, including *CHD7* (OMIM * 608892), *CREBBP*, *EVC* (OMIM * 604831), *LEF1* (OMIM + 153245), *ROR2* (OMIM * 602337), *TBX22* (OMIM * 300307) and *TP63*. Expression and functional studies have demonstrated that protein products of all these novel candidate genes play crucial roles during odontogenesis and craniofacial development [20,21,22,23,24,25,26]. Mice deficient in *Lef1*, an essential epithelial survival factor in tooth morphogenesis, show arrested tooth development at the late bud stage before forming a mesenchymal dental papilla [20,27]. Impaired tooth development and morphology were also observed in *Crebbp*, *Evc*, *Ror2* and *Trp63* (orthologue of human *TP63*) knockout mice [28,29,30,31]. Moreover, pathogenic variants of novel ns-TA candidate genes identified in this study have been reported in human clinically distinguishable syndromes and multisystem disorders associated with various craniofacial abnormalities, including anomalies in the number, size and shape, structure, position and eruption of teeth. Recently, de novo microdeletions encompassing the *LEF1* gene have also been identified in two unrelated patients with severe oligodontia and other features compatible with hypohidrotic ectodermal dysplasia [32]. In addition, monoallelic and biallelic *LEF1* variants have been proposed as causative factors for a putatively novel syndrome characterized by limb malformations and ectodermal dysplasia [33]. It is worth noting that the heterozygous *LEF1* missense variant (p.Lys95Asn) identified in the current study was found in an individual with a mild form of sporadic TA. This patient had congenital agenesis of a maxillary right lateral incisor and an abnormally shaped maxillary left lateral incisor without any other ectodermal features and structural anomalies.

Among the nucleotide alternations of these newly identified ns-TA candidate genes, three were found within sequences encoding highly conserved protein domains. One of them was the nonsense variant predicted to truncate the ROR2 receptor by 311 amino acids at the cytoplasmic kinase domain, which, together with the distal C-terminal regions, is required for receptor stability and downstream signalling [34,35]. In addition, this ROR2_p.Ser632Ter variant identified in a patient with non-syndromic hypodontia was also predicted to trigger NMD, a highly conserved RNA quality control pathway targeting mRNAs harbouring premature termination codons for degradation [36]. To date, pathogenic variants of the ROR2 receptor have been associated with Brachydactyly B (OMIM # 113000) and Robinow syndrome (OMIM # 268310), in which some individuals show dental problems such as delayed eruption and delayed root formation of permanent teeth [37]. Recent findings indicate that heterozygous recurrent nucleotide alternations, including the p.Gly559Ser missense variant located within the ROR2 kinase domain, might also be associated with isolated short stature [38].

In a male patient with the congenital lack of seven permanent teeth, a novel missense variant (p.Pro242Leu) was identified within the T-box domain of the transcription factor TBX22, acting as an essential transcriptional repressor during facial and palatal development [21]. Mutations in *TBX22* are associated with X-linked cleft palate and ankyloglossia (OMIM # 303400), which is a disorder characterized by phenotypic heterogeneity ranging from ankyloglossia only to submucous cleft palate, bifid uvula or cleft of the soft and hard palate, all with or without tongue-tie [39]. Pathogenic variants of this gene were also detected in patients with non-syndromic CLP and patients with CLP and micro/hypodontia [40,41,42]. The current study expands the phenotypic spectrum associated with *TBX22* pathogenic variants to include the ns-TA and highlights the essential role of the transcription factor TBX22 during odontogenesis. Interestingly, analyses in both humans and mice have demonstrated that during embryogenesis, *TBX22* is specifically expressed in craniofacial tissues that are affected in *TBX22* mutation carriers, including the mesenchyme of the palatal shelves, the first branchial arch, developing nose and base of the tongue [43]. High expression level in both species is also observed in the developing tooth buds [43].

Furthermore, a likely pathogenic variant for ns-TA was identified within the histone acetyltransferase (HAT) domain of CREB-binding protein (CBP), which acts as a transcriptional coactivator and epigenetic factor involved in the regulation of gene expression by modifying the chromatin structure [44]. It has been noted that the CBP HAT contains about 52% of missense mutations reported to cause Rubinstein-Taybi syndrome (OMIM # 180849), characterized by short stature, typical facial features, broad thumbs and halluces, and intellectual disability [45,46]. Dental anomalies such as talon cusps, hypodontia, supernumerary teeth and natal teeth are also frequently observed in patients with this rare autosomal dominant syndrome [44]. Animal studies have suggested that during odontogenesis, CBP provides positional information for tooth crown morphogenesis and regulates the growth of tooth cusps [22]. It is noteworthy that in our hypodontia patient with the CPB missense variant located within the HAT domain (p.Glu1560Lys), a recurrent pathogenic variant of *WNT10A* was also detected. In contrast, the second *CREBB* variant identified in the current study was the only variant fulfilling the strict project selection criteria detected in a patient with oligodontia. This novel likely pathogenic missense alteration (p.Pro344Ser) was situated outside the CBP HAT domain.

Our study provides additional evidence that ns-TA may have an oligogenic inheritance. Besides the patient harbouring variants in *WNT10A* and *CREBBP*, two other affected individuals were carriers of two pathogenic or likely pathogenic nucleotide alternations. In both cases, one variant was located within *WNT10A* while the other was within a gene not previously associated with ns-TA risk, including *EVC* and *TP63*. The heterozygous EVC_p.Ala565ValfsTer23 sequence change detected in a patient with the congenital lack of maxillary lateral incisors was categorized in the ClinVar database as pathogenic for Ellis-van Creveld syndrome (OMIM # 225500). All patients with this autosomal recessive chondrodysplasia present a wide range of dental anomalies, including hypoplastic enamel, abnormally shaped teeth, TA and premature tooth eruption [25]. Interestingly, there is evidence that heterozygous *EVC* mutations might also be associated with Weyers acrodental dysostosis (OMIM # 193530) [47]. Based on mice studies, it has been proposed that dental defects observed in patients with these congenital disorders result from disruption of the Sonic hedgehog signalling pathway combined with displaced Wnt signalling [48].

The novel TP63_p.Pro532Ala substitution together with the pathogenic WNT10A_p.Phe228Ile variant was identified in a patient with hypodontia and taurodontism of maxillary molars. In addition, one of her impacted maxillary lateral incisors was removed with complex odontoma. There is strong evidence from human and mouse studies for the TP63 transcription factor involvement in tooth development. Mutations of this gene have been associated with six autosomal dominant syndromes characterized mainly by ectodermal dysplasia, limb malformation and OFC [49]. Dental anomalies in these disorders are common and include TA, conoid teeth, taurodontism, enamel hypoplasia and dentinal dysplasia [50]. Classical animal studies have shown that p63 is an essential factor in embryonic epidermal development and epidermal keratinocyte proliferation and differentiation [51,52]. Moreover, p63 establishes enhancers at craniofacial development genes modifying their expression levels [53] and *P63*-deficient embryos fail to form ectodermal placodes that mark early tooth and hair follicle morphogenesis [30]. It should be noted that *TP63* germline mutations associated with ectodermal-related syndromes are found almost exclusively within sequences encoding functional domains of the transcription factor [54]. In contrast, the *TP63* missense variant identified in our patient is located outside these domains and does not match the rigorous project criteria used to select ns-TA risk variants. Therefore, future research will be required to approve *TP63* as a novel candidate gene for the congenital lack of teeth.

The current study also confirms the status of *WNT10A* as a key susceptibility gene for ns-TA. Its known coding variants were identified in nearly 30% of patients with a mean number of missing teeth of 9.7 (range 2 to 20). Interestingly, in our previous research conducted in an independent group of patients with ns-TA, nucleotide alternations of *WNT10A* were detected in 62% of individuals with this common dental anomaly [9]. Therefore, we can conclude, similarly to van den Boogaard et al. [55], that *WNT10A* variants may account for about 50% of all cases of hypodontia and oligodontia in the Polish population. In accordance with other reports [9,13,55,56], the most frequently observed *WNT10A* variation was p.Phe228Ile (rs121908120), which was detected in heterozygous or homozygous form in 15 individuals with variable dental phenotypes. The most severe TA was observed in homozygous carriers of this recurrent variant; however, even among them, the number of missing permanent teeth varied and ranged from 6 to 20. The presence of phenotypic variability among carriers of the same DNA lesion might be explained not only by the co-occurrence of secondary genetic events but also by epigenetic modifications or environmental factors. It is worth noting that other likely pathogenic variants have been found in six heterozygous Phe228Ile carriers, including variants of *WNT10A*, *CREBBP* and *TP63*, as described above. In ns-TA patients, the *WNT10A* alternations have been previously identified in combination with nucleotide changes of *BCOR* (OMIM * 300485), *EDA*, *EDAR*, *EDARADD* (OMIM * 606603), *LAMA3* or *LRP6* [11,12,13,14,15,16].

Thirteen pathogenic and likely pathogenic nucleotide alternations were also detected in other known susceptibility genes for ns-TA, including *AXIN2*, *EDA*, *EDAR*, *IRF6*, *LAMA3*, *LRP6*, *MSX1* and *PAX9*. The most severe dental phenotype with 15 or more missing permanent teeth was observed in female patients carrying newly discovered variants in *AXIN2*, *EDA* and *MSX1* (p.Arg675ProfsTer32, p.Asn280Lys and p.Leu123ThrfsTer52, respectively). Interestingly, the oligodontia phenotype was also present in an individual with the *IRF6* variant. The patient inherited this novel p.Arg339Gly missense substitution from her father with cleft palate only (results not shown). This result may support the hypothesis of a common genetic link between the congenital lack of teeth and orofacial clefts, which are frequently co-occurring craniofacial defects [19,57,58,59]. In addition, it may confirm the assumption that dental anomalies are subclinical phenotypes of the OFC spectrum. It should be noted that the increased incidence of dental abnormalities among unaffected family members of patients with overt clefts has already been noted [60,61].

The *IRF6* gene is one of the the key susceptibility genes susceptibility genes for OFC, of which common and rare alleles have been found etiologic in both non-syndromic and syndromic forms of this structural anomaly [62,63]. The *IRF6* deleterious variants have also been detected in patients with CLP associated with TA [19,64]. Moreover, common variants of this gene are associated with an increased risk of ns-TA [65,66]. Research in mouse models has confirmed the essential role of *Irf6* during tooth development, with a null mutation of this gene resulting in evagination of the incisor epithelium and upregulation of the canonical Wnt signalling pathway [67]. Dental epithelium-specific *Irf6* conditional knockout mice display hypodontia, occasional supernumerary incisors and molars, and alternations in the crown and root morphology [68]. In addition, their ameloblasts exhibit disturbances in adhesion, polarity and enamel formation [68].

The present study has several limitations, including the lack of segregation analysis of identified pathogenic and likely pathogenic variants in families of affected individuals. Another limitation was searching for variants underlying ns-TA only in the coding regions of a limited number of candidate genes. Since odontogenesis is a complex and not fully understood process, our criteria for selecting panel genes could cause the omission of potentially novel risk genes for ns-TA. Furthermore, the applied research approach was not intended to detect copy number variants, large deletions, duplications and insertions, and gene fusions. In addition, the in silico evaluation of the impact of the identified nucleotide changes by selected prediction tools or our variant filtering and prioritization strategy might be inappropriate, leading to the omission of clinically relevant genetic alternations. Despite its limitations, the study certainly adds to our understanding of the molecular basis of both hypodontia and oligodontia.

## 5. Conclusions

In conclusion, we have identified novel genes implicated in ns-TA, however, since mostly single nucleotide variants were detected, future research is required to confirm and fully understand their role in the aetiology of the congenital lack of permanent teeth. Additionally, our results support the importance of already known ns-TA candidate genes and provide additional evidence that ns-TA might be an oligogenic condition involving the combined effects of rare or private variants in two or more distinct genes.

## Figures and Tables

**Table 1:** Characteristics of the patient and control group.

	N	%
Tooth Agenesis (*n* = 65)		
Gender distribution		
Males	32	49.23
Females	33	50.77
Family history		
YES	36	55.39
NO	18	27.69
Unknown	11	16.92
Type of tooth agenesis ^1^		
Hypodontia (1–5 permanent teeth missing)	32	49.23
Oligodontia (≥6 permanent teeth missing)	33	50.77
Type of permanent teeth missing (total teeth missing = 469)		
Second premolar	128	27.29
Lateral incisor	109	23.24
First premolar	67	14.29
Canine	47	10.02
Central incisor	47	10.02
Second molar	45	9.60
First molar	26	5.54
Number of missing premolars	195	41.58
Number of missing incisors	156	33.26
Number of missing molars	71	15.14
Number of missing canines	47	10.02
CONTROLS (*n* = 127)		
Gender distribution		
Males	58	45.67
Females	69	54.33

In eight patients (seven with oligodontia and one with hypodontia) tooth agenesis was associated with minor ectodermal features like fine hair, dry skin or brittle nails. ^1^ Third molars were excluded from calculations.

**Table 2:** Characteristics of variants identified in patients with ns-TA.

GENE	VARIANT					In Silico Pathogenicity Prediction ^3^																CADD
Name	rs Number	DNA Change	Protein Change	Protein Domain/Repeat ^1^	Freq. ^2^	1	2	3	4	5	6	7	8	9	10	11	12	13	14	15	16	Score ^4^
**NOVEL risk genes**																						
***CHD7***	na	c.5566G>C	p.Glu1856Gln	none	no data	Dc	D	D	D	D	T	P	P	D	D	N	B	B	T	D	D	24.7
***CREBBP***	na	c.1030C>T	p.Pro344Ser	none	no data	Dc	D	D	D	D	D	P	P	T	T	D	B	B	D	T	D	24.7
***CREBBP***	na	c.4678G>A ^5^	p.Glu1560Lys	CBP/p300-type HAT domain	no data	Dc	D	D	D	D	D	P	P	D	D	D	B	P	D	D	D	29.0
***EVC***	rs753014919	c.1694delC ^5^	p.Ala565ValfsTer23	none	7.35 × 10^−5^																	na
***LEF1***	na	c.285G>C	p.Lys95Asn	none	no data	Dc	D	D	D	D	D	B	P	D	D, T	D	B	P	T	D	D	23.4
***ROR2***	na	c.1895delC	p.Ser632Ter	Protein kinase domain	no data																	na
***TBX22***	na	c.725C>T	p.Pro242Leu	T-box domain	no data	Dc	D	D	D	D	D			D	D	D	B	P		D	D	22.9
***TP63***	na	c.1594C>G ^5^	p.Pro532Ala	none	no data	Dc	D	D	N	D	T	B	P	T	T	N	B	B	T	D	D	18.8
**KNOWN risk genes**																						
***AXIN2***	na	c.2023dupC	p.Arg675ProfsTer32	none	no data																	na
***AXIN2***	na	c.2292_2302delGGTTGTCACTT	p.Val765LeufsTer24	DIX domain	no data																	na
***EDA***	CM960503	c.206G>C	p.Arg69Pro	none	no data	P	D	D		N	T			D	T, D	D, N	P	P		D	D	23.5
***EDA***	na	c.840C>A	p.Asn280Lys	none	no data	Dc	D	D		D	T			D	T, D	N	P	B		D	D	24.5
***EDA***	rs876657641	c.866G>A	p.Arg289His	none	0.00	Dc	D	D	D	D	D			D	T	N	P	P		D	D	31.0
***EDAR***	na	c.256A>C	p.Ile86Leu	TNFR-Cys repeat	no data	Dc	D	D	D	D	T	P	P	T	D	N	P	P	D	D	D	24.6
***IRF6***	na	c.1015A>G	p.Arg339Gly	none	no data	Dc	D	D	D	D	D	P	P	D	D	D	P	P	T	D	D	28.7
***LAMA3***	rs771405735	c.916G>T	p.Glu306Ter	Laminin EGF-like domain	0.00	Dc		D	N			P	P						T			38.0
***LRP6***	na	c.1418G>C	p.Arg473Pro	LDL-receptor class B repeat	no data	Dc	D	D	D	D	D	P	P	T	D	D	B	P	D	D	D	25.0
***LRP6***	na	c.1629C>G	p.Asp543Glu	LDL-receptor class B repeat	no data	Dc	D	D	N	D	T, D	B	B	D	T	D	B	B	T	T	D	21.2
***LRP6***	na	c.1735A>G	p.Lys579Glu	LDL-receptor class B repeat	no data	Dc	D	D	D	D	D, T	P	P	D	D	D	P	P	T	D	D	24.8
***MSX1***	na	c.365dupG	p.Leu123ThrfsTer52	none	no data																	na
***PAX9***	na ^6^	c.406C>T	p.Gln136Ter	none	no data	Dc		D	N	D		P	P						T			39.0
***WNT10A***	rs121908119	c.321C>A	p.Cys107Ter	none	1.60 × 10^−3^	Dc		D	N	D		P	P						T			35.0
***WNT10A***	rs141074983	c.337C>T	p.Arg113Cys	none	2.65 × 10^−4^	Dc	T	D	D		D	B	B	D	D	D	B	B	T	T	T	16.2
***WNT10A***	rs143424659	c.668G>A	p.Arg223His	none	0.00	Dc	T	D	N	D	T	B	P	D	D	N	B	B	D	D	D	22.8
***WNT10A***	rs121908120	c.682T>A	p.Phe228Ile	none	2.23 × 10^−2^	Dc	D	D	D	D	D	P	P	D	D	D	P	P	T	D	D	28.4
***WNT10A***	rs772154760	c.695G>A	p.Arg232Gln	none	1.47 × 10^−5^	Dc	T	D	D	D	D	P	P	D	D	D	P	B	D	D	D	31.0
***WNT10A***	rs1553623335	c.1034T>C	p.Phe345Ser	none	no data	Dc	D	D	D	D	D	P	P	D	D	D	P	P	D	D	D	33.0

^1^ The Universal Protein Resource (UniProt). https://www.uniprot.org/ (access on 1 March 2022). ^2^ Frequency. The Genome Aggregation Database v.3.1.1 [gnomAD, genome sequencing data, population: European (non-Finnish)]. https://gnomad.broadinstitute.org/ (access on 1 March 2022). ^3^ Pathogenicity of missense variants was predicted using 16 in silico tools (1—MutationTaster, 2—FATHMM, 3—FATHMM-MKL, 4—FATHMM-XF, 5—LRT, 6—DEOGEN2, 7—EIGEN, 8—EIGEN PC, 9—SIFT, 10—SIFT4G, 11—PROVEAN, 12—MVP, 13—REVEL, 14—PrimateAI, 15—MetaSVM and 16—MetaLR). B—benign, D—damaging/deleterious, Dc—disease causing, N—neutral, P—pathogenic, T—tolerated. ^4^ CADD—Combined Annotation Dependent Depletion version 1.6. https://cadd.gs.washington.edu/ (access on 1 March 2022). ^5^ Variants identified in patients harbouring the WNT10A_p.Arg113Cys or WNT10A_p.Phe228Ile. ^6^ Variant described previously in a Finnish family with non-syndromic oligodontia [13]. na, not available.

**Table 3:** Dental phenotype of patients with variants identified in genes other than *WNT10A*.

					Right Upper Jaw (q1)							Left Upper Jaw (q2)						
					17	16	15	14	13	12	11	21	22	23	24	25	26	27
		Family	Number of		47	46	45	44	43	42	41	31	32	33	34	35	36	37
Patient	Gender	History	Missing Teeth ^1^	Identified Variant ^2^	Right Lower Jaw (q4)							Left Lower Jaw (q3)						
NOVEL candidate genes																		
TA_1	Female	Yes	7	CBP_p.Pro344Ser_HET			X	X							X			
							X	X							X	X		
TA_2	Male	No	7	TBX22_p.Pro242Leu_HEMI			X									X		
							X				X	X			X	X		
TA_3	Female	Yes	5	ROR2_p.Ser632Ter_HET	X													X
					X		X									X		
TA_4	Male	na	2	CHD7_p.Glu1856Gln_HET						X			X					
TA_5	Female	No	2	LEF1_p.Lys95Asn_HET						X			p					
KNOWN candidate genes																		
TA_6	Female	Yes	24	EDA_p.Asn280Lys_HET	X	X	X	X		X			X		X	X	X	X
					X	X	X	X	X	X	X	X	X	X	X	X	X	X
TA_7	Female	Yes	21	AXIN2_p.Arg675ProfsTer32_HET		X	X	X	X	X			X	X	X	X	X	
					X	X	X	X		X	X	X	X			X	X	X
TA_8	Female	Yes	15	MSX1_p.Leu123ThrfsTer52_HET			X	X		X			X		X	X		
					X	X	X				X	X			X	X	X	X
TA_9	Female	No	13	LRP6_Lys579Glu_HET	X		X	X		X			X	X		X		X
					X		X	X								X		X
TA_10	Female	No	10	IRF6_p.Arg339Gly_HET ^3^			X	X		X			X		X	X		
							X		X							X	X	
TA_11	Male	na	10	LRP6_p.Asp543Glu_HET			X		X	X			X	X				
							X		X				X	X	X			
TA_12	Male	No	7	AXIN2_p.Val765LeufsTer24_HET						X			X					
					X					X	X	X	X					
TA_13	Female	Yes	7	LAMA3_p.Glu306Ter_HET	X		X									X		X
										X			X			X		
TA_14	Female	Yes	6	LRP6_p.Arg473Pro_HET					X	X			X	X		X		
							X											
TA_15 ^4^	Male	Yes	6	PAX9_p.Gln136Ter_HET		X		X		X								
												X	X	X				
TA_16	Male	Yes	5	EDA_p.Arg69Pro_HEMI						X			X					
										X	X	X						
TA_17	Male	Yes	5	EDA_p.Arg289His_HEMI					X	X			X					
											X	X						
TA_18	Male	Yes	4	EDA_p.Arg289His_HEMI		X												
						X			X							X		
TA_19	Male	na	2	EDAR_p.Ile86Leu_HET						X			X					

In patients 4, 11 and 15, tooth agenesis was associated with some features of ectodermal dysplasia. Missing teeth are indicated with X; p, peg-shaped second incisor. The CREB-binding protein (CBP) is encoded by the *CREBBP* gene. ^1^ Number of missing teeth excluding third molars. ^2^ HEMI, variant identified in hemizygous form (chromosome X); HET, variant identified in heterozygous form. ^3^ Nucleotide variant inherited from father with cleft palate. ^4^ The patient’s younger brother had a cleft lip and palate.

**Table 4:** Dental phenotype of patients with *WNT10A* gene variants.

					Right Upper Jaw (q1)							Left Upper Jaw (q2)						
					17	16	15	14	13	12	11	21	22	23	24	25	26	27
		Family	Number of		47	46	45	44	43	42	41	31	32	33	34	35	36	37
Patient	Gender	History	Missing Teeth ^1^	Identified Variant ^2^	Right Lower Jaw (q4)							Left Lower Jaw (q3)						
TA_20	Female	na	20	WNT10A_p.Phe228Ile_HOM		X	X	X	X	X			X	X	X	X	X	
					X	X		X	X		X	X		X	X	X	X	
TA_21	Male	Yes	19	WNT10A_p.Phe228Ile_HET + WNT10A_p.Phe345Ser_HET			X	X	X	X			X	X	X	X		
					X		X		X	X	X	X	X	X	X	X		X
TA_22	Male	No	16	WNT10A_p.Cys107Ter_HET + WNT10A_p.Phe228Ile_HET			X		X	X			X	X	X	X		
							X	X	X	X	X	X	X	X		X		
TA_23	Male	Yes	16	WNT10A_p.Phe228Ile_HOM	X		X	X					X		X	X		X
					X		X	X		X	X		X		X	X		X
TA_24	Female	Yes	14	WNT10A_p.Phe228Ile_HOM		X	X	X		X			X		X	X	X	
						X	X	X							X	X	X	
TA_25	Female	No	14	WNT10A_p.Phe228Ile_HOM		X	X	X		X			X		X	X		
					X			X		X	X	X			X			X
TA_26	Female	Yes	12	WNT10A_p.Phe228Ile_HOM	X		X	X					X		X	X		X
					X		X					X				X		X
TA_27	Male	Yes	10	WNT10A_p.Phe228Ile_HOM					X	X			X	X				
									X	X	X	X	X	X				
TA_28	Female	No	9	WNT10A_p.Arg113Cys_HET + WNT10A_p.Phe228Ile_HET			X	X	X					X	X			
							X	X							X	X		
TA_29	Female	Yes	9	WNT10A_p.Phe228Ile_HET			X	X		X			X		X	X		
							X								X	X		
TA_30	Male	Yes	8	WNT10A_p.Cys107Ter_HET			X	X							X	X		
							X	X							X	X		
TA_31	Male	No	6	WNT10A_p.Phe228Ile_HOM		X							X					
										X	X	X	X					
TA_32	Female	Yes	5	WNT10A_p.Arg232Gln_HET						X			X					
							X				X	X						
TA_33	Female	No	5	WNT10A_p.Phe228Ile_HET + TP63_p.Pro532Ala_HET			X		X	p						X		
							X									X		
TA_34	Female	Yes	4	WNT10A_p.Phe228Ile_HET + CBP_p.Glu1560Lys_HET						p								
					X		X									X		X
TA_35	Female	Yes	3	WNT10A_p.Phe228Ile_HET				X		X			X					
TA_36	Female	Yes	2	WNT10A_p.Arg113Cys_HET + EVC_p.Ala565ValfsTer23_HET						X			X					
TA_37	Male	No	2	WNT10A_p.Arg223His_HET + WNT10A_p.Phe228Ile_HET						X			X					

In patients number 21, 27, 28 and 29, tooth agenesis was associated with some features of ectodermal dysplasia. Patient number 33 was diagnosed with compound odontoma at the age of 12. Missing teeth are indicated with X; p, peg-shaped lateral incisor. The CREB-binding protein (CBP) is encoded by the *CREBBP* gene. ^1^ Number of missing teeth excluding third molars. ^2^ HET, variant identified in heterozygous form; HOM, variant identified in homozygous form.

**Table 5:** Association of common exonic variants with the risk of ns-TA.

				MAF Cases		
Gene	Variant ID	Protein Effect ^1^	Alleles ^2^	/MAF Controls	P_trend_ value	OR (95%CI)
***AXIN2***	rs2240308	p.Pro50Ser	C/T	0.48 (C)/0.46 (T)	3.17 × 10^−1^	1.24 (0.82–1.90)
***AXIN2***	rs9915936	p.Pro455Pro	A/G	0.17 (A)/0.12 (A)	1.90 × 10^−1^	1.49 (0.82–2.71)
***AXIN2***	rs1133683	p.Pro462Pro	C/T	0.41 (C)/0.38 (C)	5.89 × 10^−1^	1.12 (0.73–1.72)
***EDAR***	rs260632	p.Ser250Ser	C/T	0.13 (C)/0.11 (C)	6.68 × 10^−1^	1.15 (0.61–2.17)
***EDAR***	rs12623957	p.Cys352Cys	C/T	0.16 (C)/0.17 (C)	7.12 × 10^−1^	1.11 (0.63–1.96)
***EVC***	rs35870680	p.Ser83Ser	A/G	0.17 (G)/0.16 (G)	6.66 × 10^−1^	1.13 (0.64–1.98)
***EVC***	rs6414624	p.Tyr258His	T/C	0.24 (T)/0.21 (T)	5.13 × 10^−1^	1.19 (0.72–1.95)
***EVC***	rs4688963	p.Asn323Asn	T/C	0.23 (C)/0.32 (C)	4.90 × 10^−2^	1.62 (1.00–2.63)
***EVC***	rs4688962	p.Leu342Leu	G/C	0.29 (C)/0.37 (C)	1.18 × 10^−1^	1.43 (0.91–2.25)
***EVC***	rs33929747	p.Leu356Leu	A/G	0.40 (G)/0.37 (G)	4.99 × 10^−1^	1.16 (0.75–1.79)
***EVC***	rs2302075	p.Thr449Lys	C/A	0.31 (C)/0.24 (C)	1.50 × 10^−1^	1.43 (0.89–2.28)
***EVC***	rs1383180	p.Arg576Gln	G/A	0.41 (A)/0.45 (A)	4.48 × 10^−1^	1.18 (0.77–1.80)
***EVC***	rs11737221	p.Gly618Gly	C/T	0.27 (T)/0.32 (T)	3.76 × 10^−1^	1.23 (0.77–1.95)
***IRF6***	rs2013162	p.Ser153Ser	G/T	0.40 (T)/0.30 (T)	3.29 × 10^−2^	1.60 (1.03–2.49)
***LAMA3***	rs9962023	p.Ala967Ala	T/C	0.36 (T)/0.32 (T)	4.32 × 10^−1^	1.20 (0.77–1.86)
***LAMA3***	rs867449	p.Gly1420Gly	G/C	0.42 (G)/0.42 (G)	9.54 × 10^−1^	1.01 (0.66–1.55)
***LAMA3***	rs12965685	p.Pro1510Pro	C/T	0.42 (C)/0.42 (C)	8.94 × 10^−1^	1.03 (0.67–1.58)
***LAMA3***	rs1154226	p.Ala2049Ala	C/G	0.33 (G)/0.31 (G)	7.11 × 10^−1^	1.09 (0.69–1.71)
***LAMA3***	rs1154232	p.Asn2815Lys	C/A	0.26 (A)/0.23 (A)	5.86 × 10^−1^	1.15 (0.70–1.87)
***LAMA3***	rs1131521	p.Leu2911Leu	C/T	0.26 (T)/0.23 (T)	5.86 × 10^−1^	1.15 (0.70–1.87)
***LRP6***	rs2302685	p.Val1062Ile	G/A	0.18 (G)/0.22 (G)	4.29 × 10^−1^	1.24 (0.73–2.12)
***MSX1***	rs36059701	p.Ala40Gly	C/G	0.14 (G)/0.14 (G)	8.72 × 10^−1^	1.05 (0.58–1.92)
***PAX9***	rs12881240	p.His239His	C/T	0.27 (C)/0.37 (C)	7.58 × 10^−2^	1.54 (0.97–2.44)
***PAX9***	rs4904210	p.Ala240Pro	G/C	0.27 (G)/0.19 (G)	6.95 × 10^−2^	1.59 (0.96–2.62)
***ROR2***	rs10820900	p.Thr245Ala	A/G	0.31 (A)/0.31 (A)	9.42 × 10^−1^	1.02 (0.65–1.60)
***ROR2***	rs10761129	p.Val819Ile	G/A	0.27 (G)/0.32 (G)	2.44 × 10^−1^	1.32 (0.83–2.11)
***ROR2***	rs10992063	p.Tyr696Tyr	C/T	0.48 (T)/0.44 (T)	4.93 × 10^−1^	1.16 (0.76–1.77)
***ROR2***	rs2230577	p.Pro718Pro	C/T	0.12 (T)/0.10 (T)	4.89 × 10^−1^	1.26 (0.65–2.46)
***WNT10A***	**rs121908120**	**p.Phe228Ile**	**T/A**	**0.16 (A)/0.004 (A)**	**9.15 × 10^−7^**	**47.86 (6.36–360.45)**

None common (MAF ≥ 0.1 in the case group) exonic and splicing variants were identified in *CHD7*, *CREBBP*, *EDA*, *LEF1*, *TBX22* and *TP63*. MAF, minor allele frequency; OR, Odds Ratio; CI, confidence interval. *p*_trend_-values significant after the Boferroni correction are highlighted in bold font [*p* < 1.72 × 10^−3^ (0.05/29 tested variants)]. ^1^ Based on the MANE (Matched Annotation from NCBI and EMBL-EBI) Select transcript. ^2^ Underline denotes the risk allele.

## Data Availability

The de-identified datasets generated through this study can be provided by the corresponding author upon request.

## Supplementary Materials

The following supporting information can be downloaded at: https://www.mdpi.com/article/10.3390/jcm11206089/s1, Table S1: NGS targeted multigene panel for ns-TA; Table S2: Sequencing template PCR conditions and sequencing primers; Table S3: Characteristics of genes harbouring rare variants identified in patients with ns-TA (gene-phenotype relationships); Figure S1: Detection of a novel *CHD7* missense variant (p.Glu1856Gln); Figure S2: Detection of a novel *CREBBP* missense variant (p.Pro344Ser); Figure S3: Detection of a novel *LEF1* missense variant (p.Lys95As); Figure S4: Detection of a novel *ROR2* nonsense variant (p.Ser632Ter); Figure S5: Detection of a novel *TBX22* missense variant (p.Pro242Leu); Figure S6: Detection of a novel *CREBBP* missense variant (p.Glu1560Lys); Figure S7: Detection of a known *EVC* frameshift variant (Ala565ValfsTer23); Figure S8: Detection of a novel *TP63* missense variant (p.Pro532Ala).
