# Supplementary material for: Novel Candidate Genes for Non-Syndromic Tooth Agenesis Identified Using Targeted Next-Generation Sequencing
Source: J Clin Med. 2022 Oct 15;11(20):6089. doi: 10.3390/jcm11206089 (PMC9605476; doi:10.3390/jcm11206089)
Supplement: Supplementary file 1 [file jcm-11-06089-s001.zip › jcm-1958592-Supplementary-revised-1st/Supplementary figures 1-8_R1.pdf]

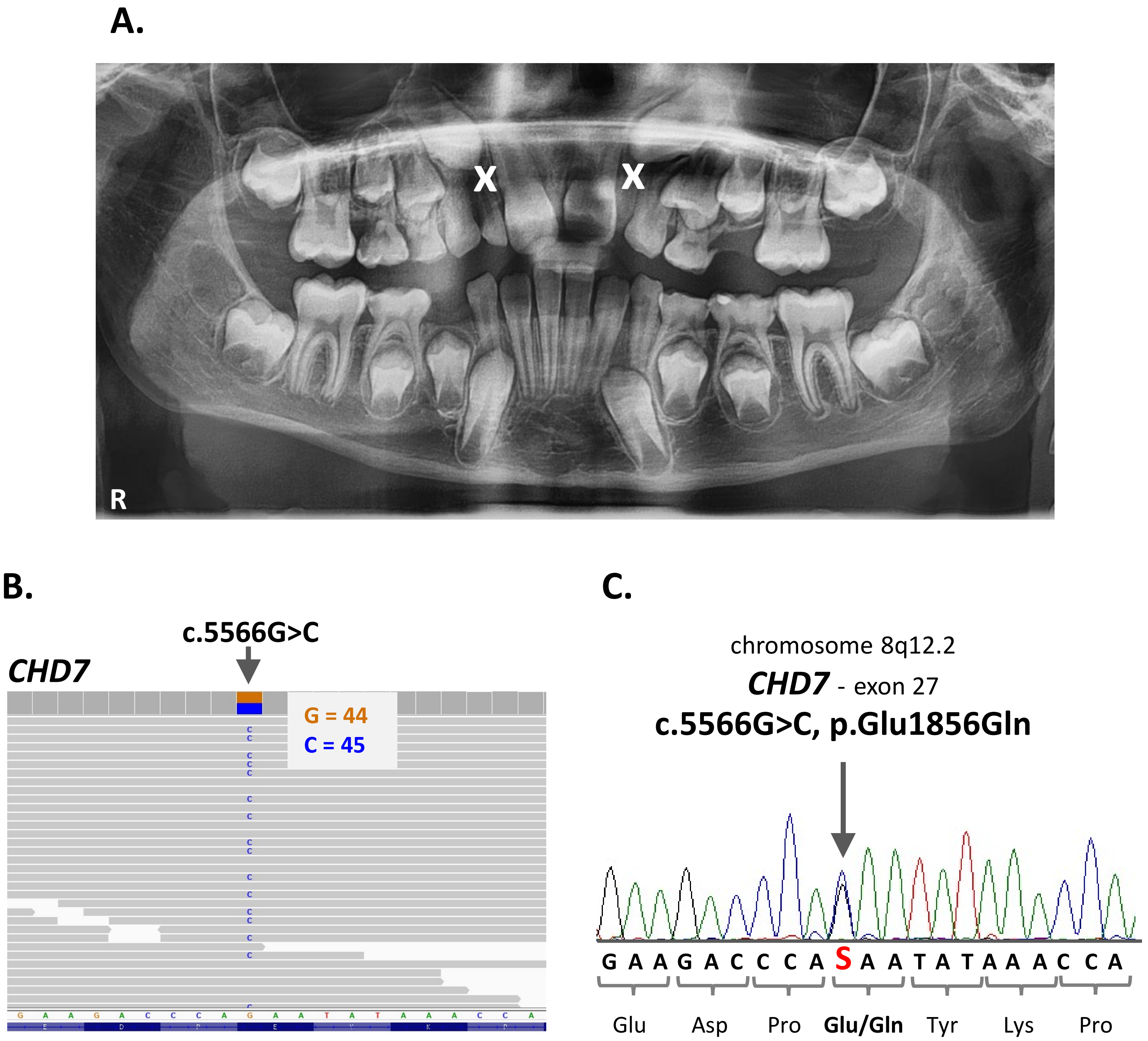


**Supplementary Figure 1**

**Detection of a novel *CHD7* missense variant (p.Glu1856Gln)**

In a patient with the congenital lack of maxillary lateral incisors (A), targeted next-generation sequencing identified a novel likely pathogenic variant in exon 27 of the *CHD7* gene (B). The presence of this heterozygous c.5566G>C transversion leading to p.Glu1856Gln substitution was confirmed by Sanger sequencing (C). On the panoramic radiograph, missing permanent teeth are indicated with X; R, right.

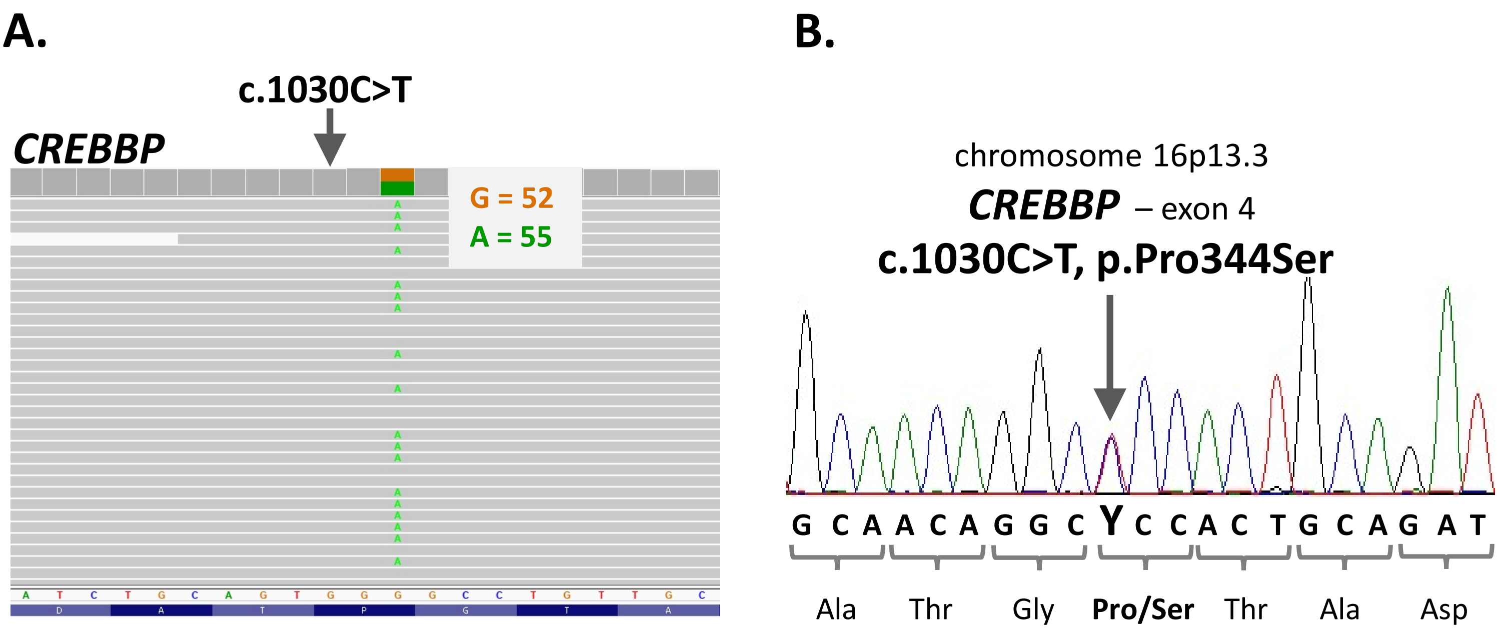


**Supplementary Figure 2**

**Detection of a novel *CREBBP* missense variant (p.Pro344Ser)**

In a patient with the congenital lack of maxillary and mandibular first premolars, maxillary right second premolar and mandibular second premolars (the C not available), targeted next-generation sequencing identified a novel likely pathogenic variant in exon 4 of the *CREBBP* gene (B). The presence of this heterozygous c.1030C>T transition leading to p.Pro344Ser substitution was confirmed by Sanger sequencing (C).

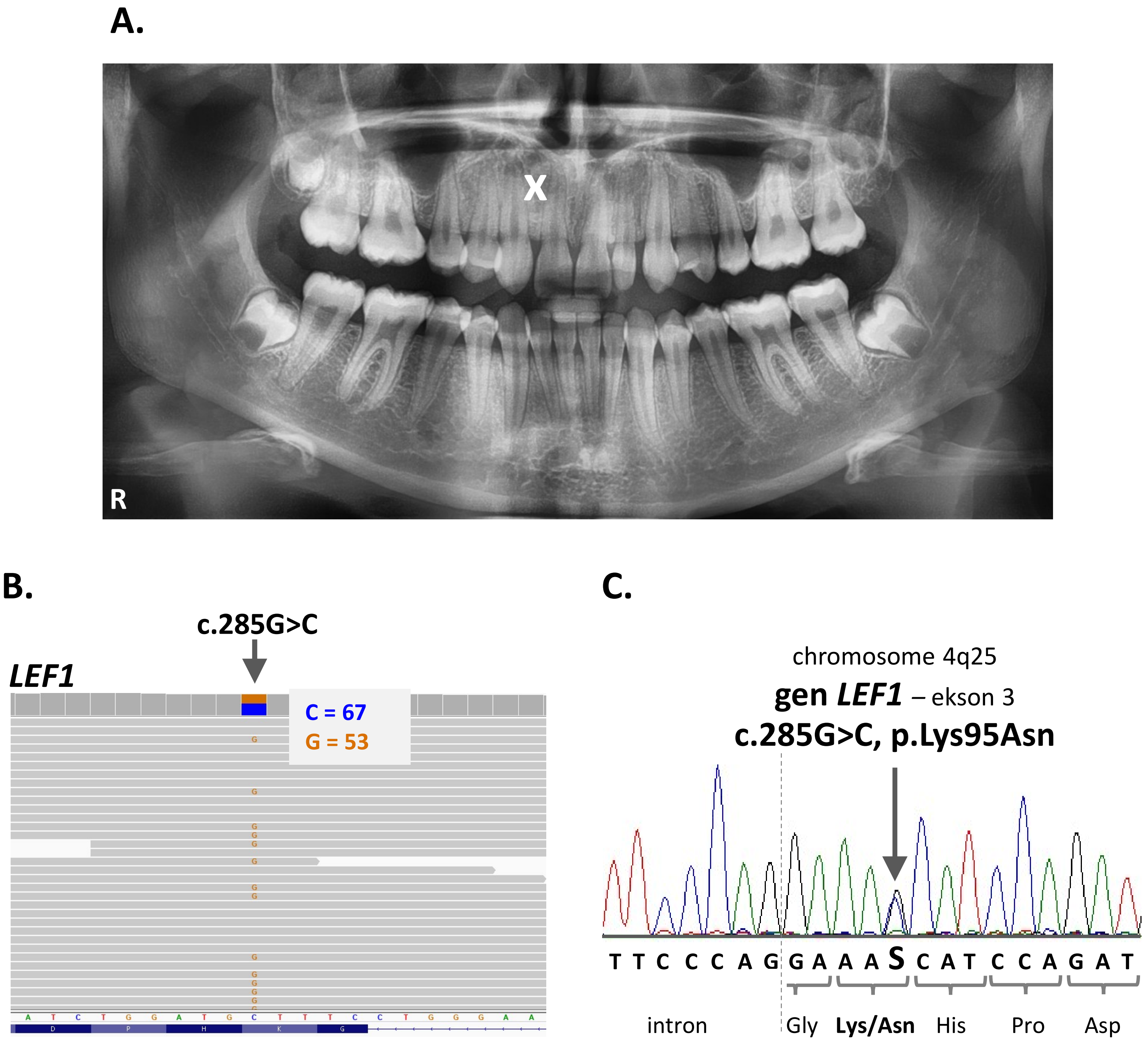


**Supplementary Figure 3**

**Detection of a novel *LEF1* missense variant (p.Lys95As)**

In a patient with the congenital lack of maxillary right lateral incisor and a peg-shaped maxillary left lateral incisor (A), targeted next-generation sequencing identified a novel likely pathogenic variant in exon 3 of the *LEF1* gene (B). The presence of this heterozygous transversion c.285G>C leading to p.Lys95Asn substitution was confirmed by Sanger sequencing (C). On the panoramic radiograph, missing permanent teeth are indicated with X; R, right.

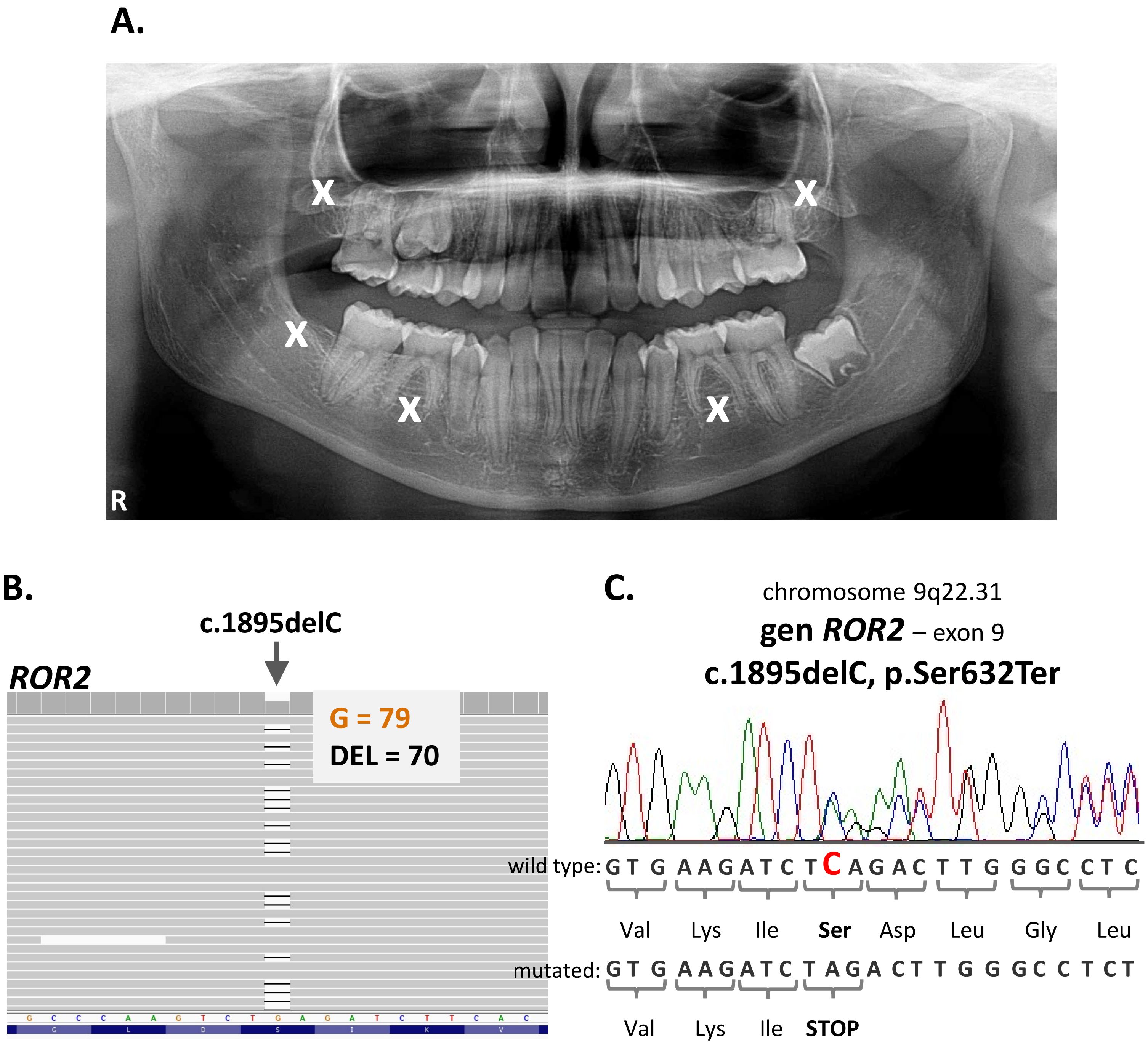


**Supplementary Figure 4**

**Detection of a novel *ROR2* nonsense variant (p.Ser632Ter)**

In a patient with the congenital lack of maxillary second molars, mandibular second premolars and mandibular right second molar (A), targeted next-generation sequencing identified a novel likely pathogenic variant in exon 9 of the *ROR2* gene (B). The presence of this heterozygous c.1895delC deletion introducing a premature STOP codon (p.Ser632Ter) was confirmed by Sanger sequencing (C). The identified deletion is located within a sequence encoding the protein kinase domain of the ROR2 receptor. On the panoramic radiograph, missing permanent teeth are indicated with X; R, right.

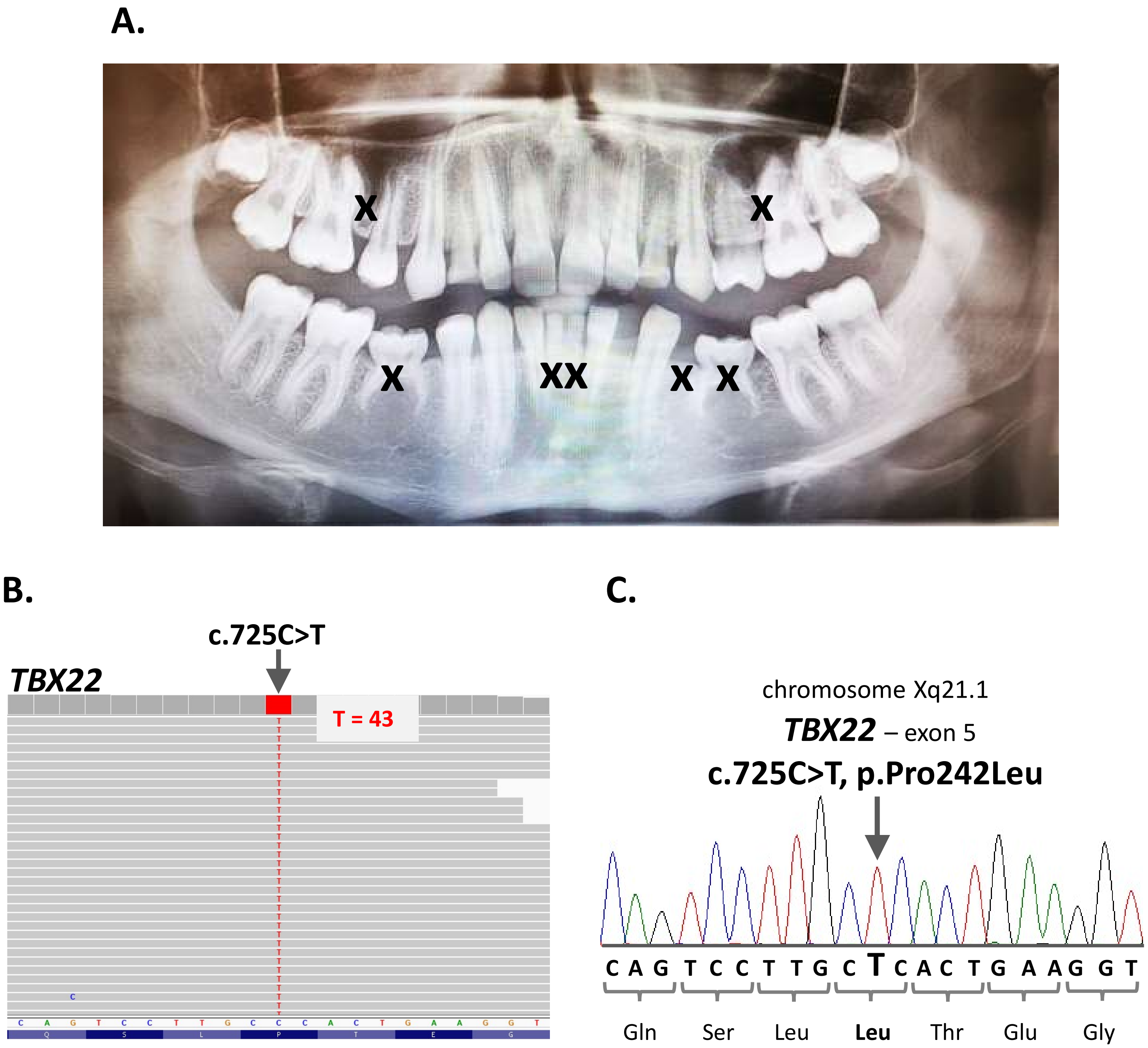


**Supplementary Figure 5**

**Detection of a novel *TBX22* missense variant (p.Pro242Leu)**

In a patient with the congenital lack of maxillary and mandibular second premolars, mandibular central incisors and mandibular left first premolar (A), targeted next-generation sequencing identified a novel likely pathogenic variant in exon 5 of the *TBX22* gene (B). The presence of this hemizygous c.725C>T transition leading to p.Pro242Leu substitution was confirmed by Sanger sequencing (C). The identified variant is located within a sequence encoding the T-box domain of the transcription factor TBX22. On the panoramic radiograph, missing permanent teeth are indicated with X; R, right.

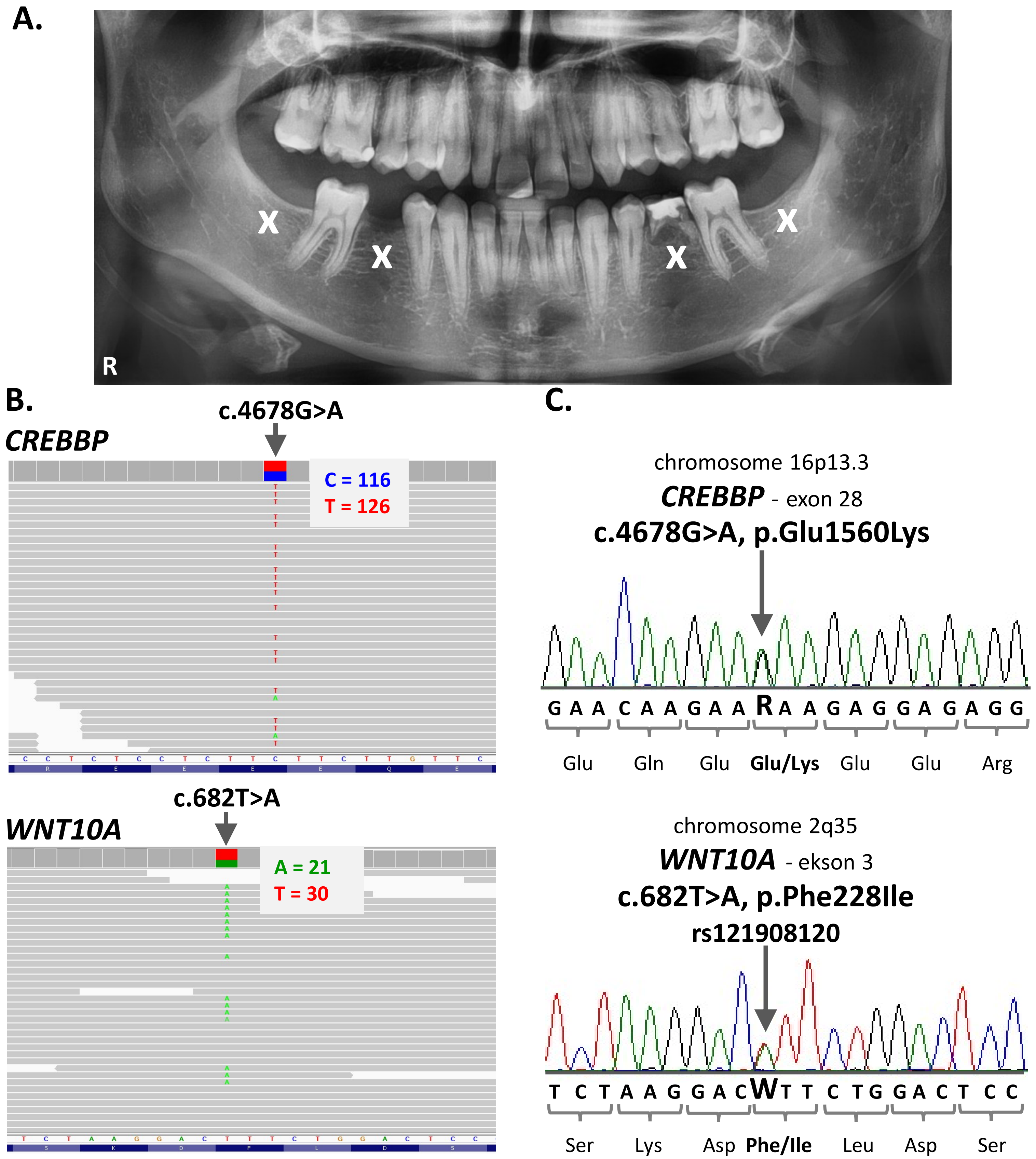


**Supplementary Figure 6**

**Detection of a novel *CREBBP* missense variant (p.Glu1560Lys)**

In a patient with the congenital lack of mandibular second premolars and second molars, and a peg-shaped maxillary right lateral incisor (A), targeted next-generation sequencing identified a novel likely pathogenic variant in exon 28 of the *CREBBP* gene and a known pathogenic variant in exon 3 of the *WNT10A* gene (B). The presence of these heterozygous nucleotide alternations was confirmed by Sanger sequencing (C). The c.4678G>A transition leading to p.Glu1560Lys substitution is located within a sequence encoding the CBP/p300-type HAT domain of the CREBBP protein. The *WNT10A* c.682T>A transversion (rs121908120) results in p.Phe228Ile change. On the panoramic radiograph, missing permanent teeth are indicated with X; R, right.

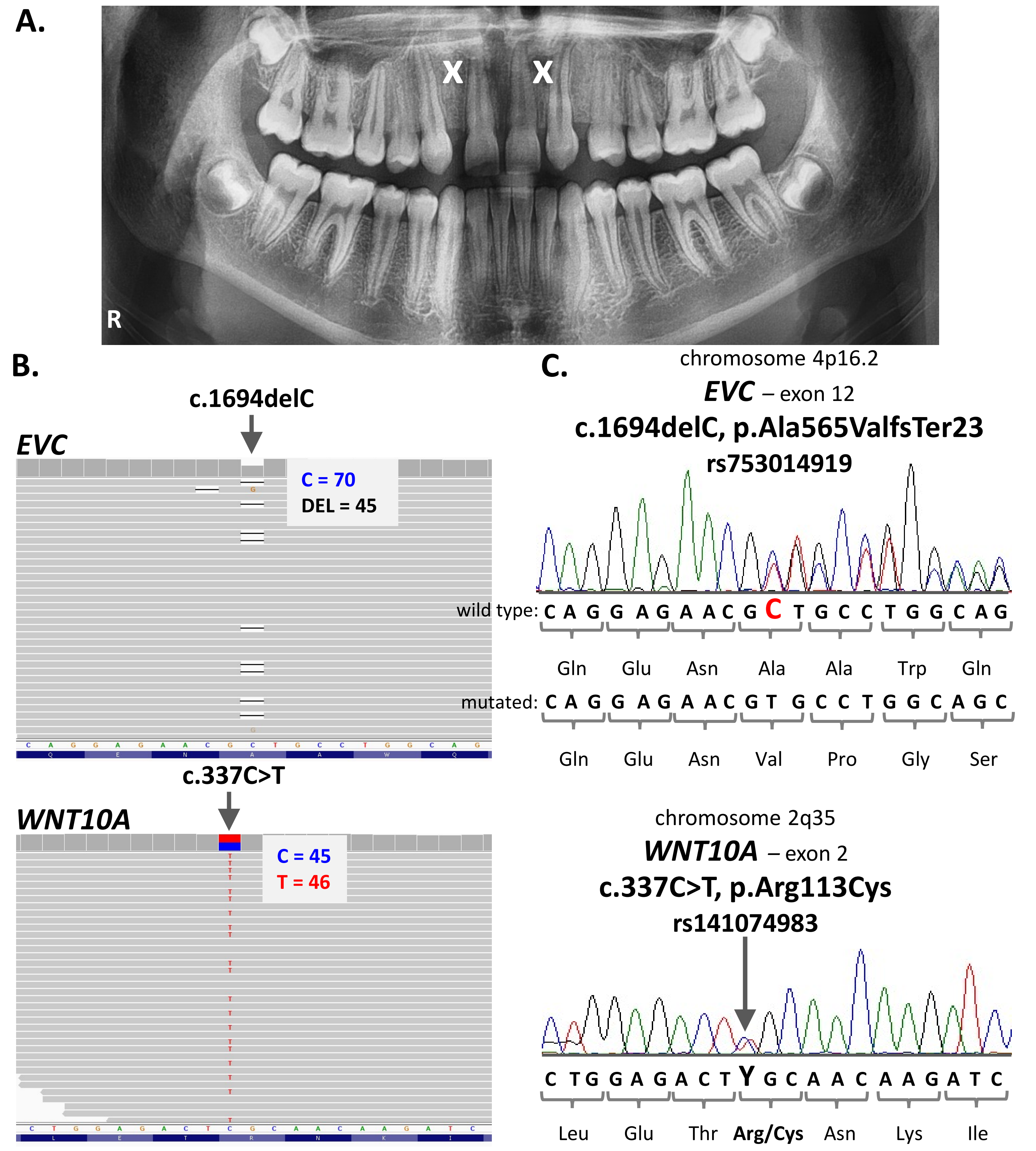


**Supplementary Figure 7**

**Detection of a known *EVC* frameshift variant (Ala565ValfsTer23).**

In a patient with the congenital lack of maxillary lateral incisors (A), targeted next-generation sequencing identified a likely pathogenic nucleotide variant in exon 12 of the *EVC* gene and exon 2 of the *WNT10A* gene (B). The presence of these known heterozygous nucleotide alternations (rs753014919 and rs141074983, respectively) was confirmed by Sanger sequencing (C). The *EVC* c.1694delC deletion leads to a frameshift and introduces a premature STOP codon (p.Ala565ValfsTer23), while the *WNT10A* c.337C>T transition results in p.Arg113Cys substitution. On the panoramic radiograph, missing permanent teeth are indicated with X; R, right.

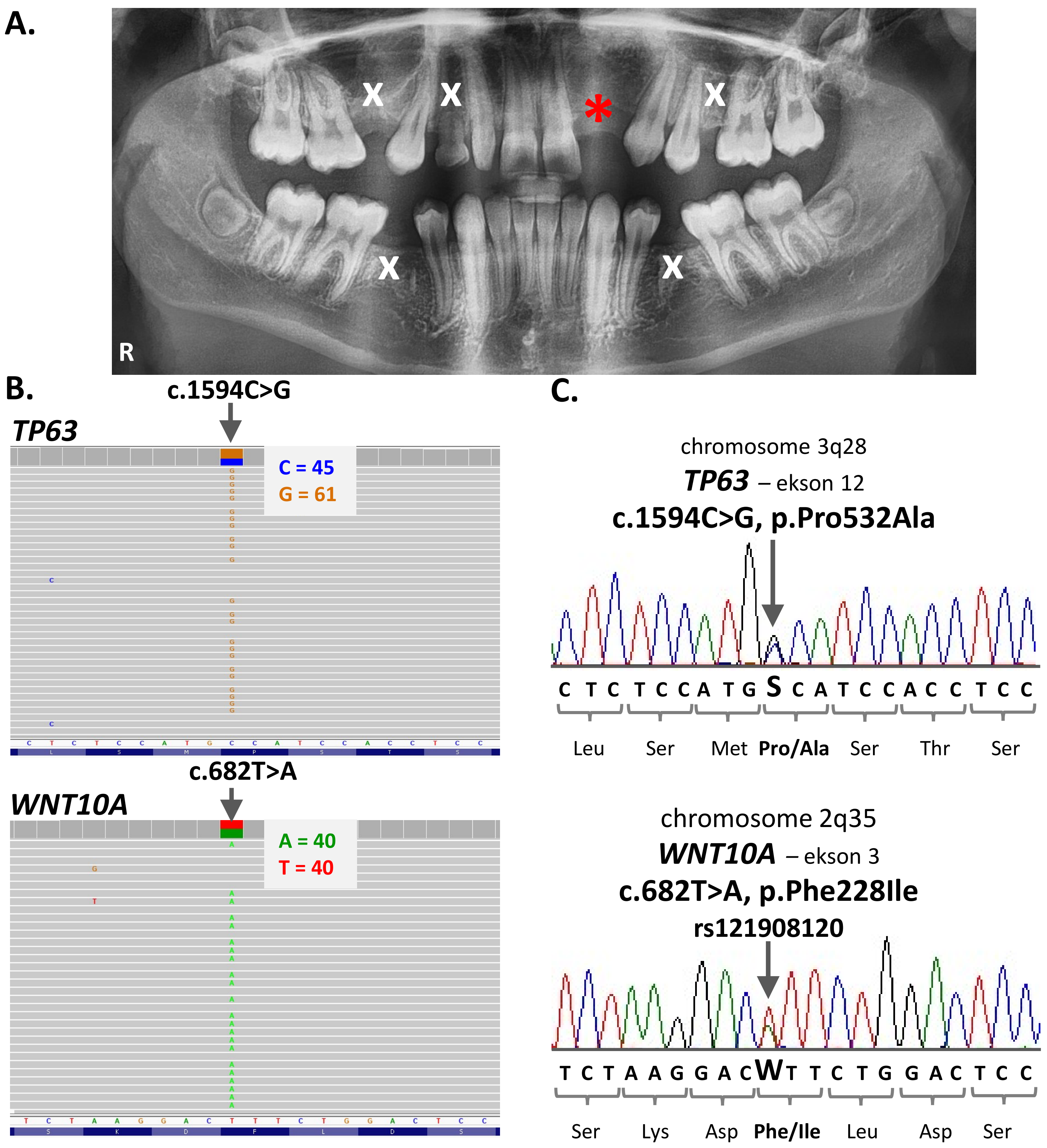


**Supplementary Figure 8**

**Detection of a novel *TP63* missense variant (p.Pro532Ala).**

In a patient with the congenital lack of maxillary and mandibular second premolars and maxillary right canine (A), targeted next-generation sequencing identified a novel likely pathogenic variant in exon 12 of the *TP63* gene and a known pathogenic variant in exon 3 of the *WNT10A* gene (B). The presence of these heterozygous nucleotide alternations was confirmed by Sanger sequencing (C). The *TP63* c.1594C>G transversion leads to p.Pro532Ala substitution, while the *WNT10A* c.682T>A transversion (rs121908120) results in p.Phe228Ile change. On the panoramic radiograph, missing permanent teeth are indicated with X; impacted maxillary left lateral incisor (*) was removed together with complex odontoma; in addition, taurodontism of maxillary first and second molars and abnormally shaped maxillary right lateral incisor are observed; R, right.
