# Supplementary figures and images for: Novel Candidate Genes for Non-Syndromic Tooth Agenesis Identified Using Targeted Next-Generation Sequencing
Source: J Clin Med. 2022 Oct 15;11(20):6089. doi: 10.3390/jcm11206089 (PMC9605476; doi:10.3390/jcm11206089)

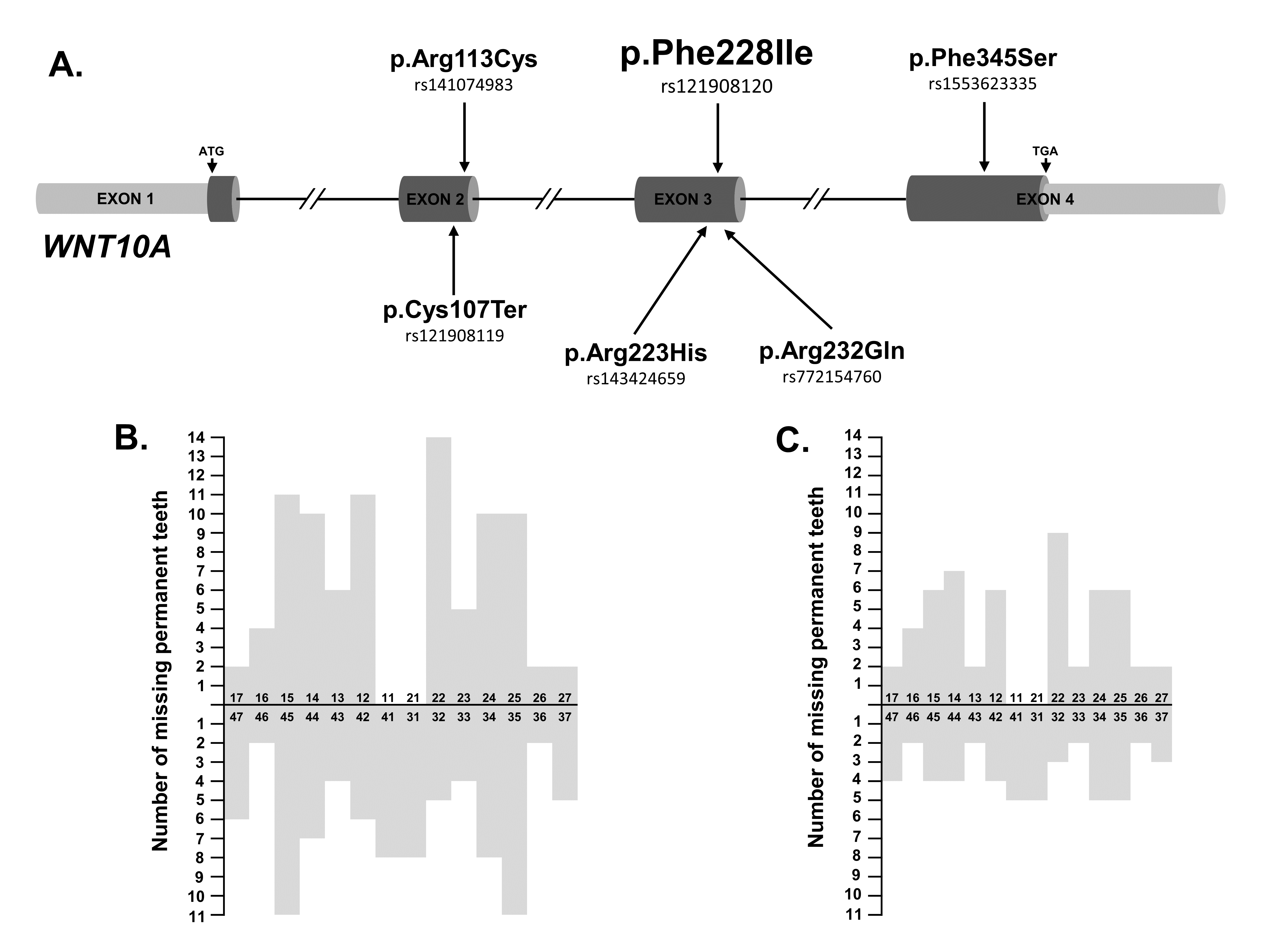

Supplement: Supplementary file 1 [file jcm-11-06089-s001.zip › jcm-1958592-Supplementary-revised-1st/Figure 1_R1.png]
